# Identification of Protein Markers in Patients Infected with *Plasmodium knowlesi*, *Plasmodium falciparum* and *Plasmodium vivax*

**DOI:** 10.3390/ijms151119952

**Published:** 2014-11-03

**Authors:** Alan Kang-Wai Mu, Ping Chong Bee, Yee Ling Lau, Yeng Chen

**Affiliations:** 1 Department of Oral Biology and Biomedical Sciences, Faculty of Dentistry, University of Malaya, Kuala Lumpur 50603, Malaysia; E-Mail: alanmukangwai@yahoo.com; 2 Department of Medicine, Faculty of Medicine, University of Malaya, Kuala Lumpur 50603, Malaysia; E-Mail: pcbee@um.edu.my; 3 Department of Parasitology, Faculty of Medicine, University of Malaya, Kuala Lumpur 50603, Malaysia; E-Mail: lauyeeling@um.edu.my; 4 Oral Cancer Research and Coordinating Centre, Faculty of Dentistry, University of Malaya, Kuala Lumpur 50603, Malaysia

**Keywords:** malaria, plasmodium, iTRAQ

## Abstract

Malaria is caused by parasitic protozoans of the genus *Plasmodium* and is one of the most prevalent infectious diseases in tropical and subtropical regions. For this reason, effective and practical diagnostic methods are urgently needed to control the spread of malaria. The aim of the current study was to identify a panel of new malarial markers, which could be used to diagnose patients infected with various *Plasmodium* species, including *P. knowlesi*, *P. vivax* and *P. falciparum*. Sera from malaria-infected patients were pooled and compared to control sera obtained from healthy individuals using the isobaric tags for relative and absolute quantitation (iTRAQ) technique. Mass spectrometry was used to identify serum proteins and quantify their relative abundance. We found that the levels of several proteins were increased in pooled serum from infected patients, including cell adhesion molecule-4 and C-reactive protein. In contrast, the serum concentration of haptoglobin was reduced in malaria-infected individuals, which we verified by western blot assay. Therefore, these proteins might represent infectious markers of malaria, which could be used to develop novel diagnostic tools for detecting *P. knowlesi*, *P. vivax* and *P. falciparum*. However, these potential malarial markers will need to be validated in a larger population of infected individuals.

## 1. Introduction

Malaria has been reported to affect over two billion people globally, impacting numerous countries. In fact, according to the World Health Organization’s World Malaria Report 2013 and the Global Malaria Action Plan, there are currently 3.4 billion people at risk of malaria infection in 97 countries and territories [1]. It is caused by protozoan parasites belonging to the genus *Plasmodium*. Among the *Plasmodium* species, *P. falciparum*, *P. vivax*, *P. knowlesi*, *P. malariae* and *P. ovale* are known to infect humans under natural conditions [2,3]. Collectively, these *Plasmodium* species are known as the human malaria species. However, *P. malariae* and *P. ovale* are rare and less dangerous compared to other species [2].

There are various perceptions regarding the source of malaria. While midwives and pregnant women consider mosquitoes to be the main transmitters of malaria in East Sudan [4], Nigerian locals indicate that they are unsure of the exact cause of malaria. The various *Plasmodium* species display distinct characteristics. *P. knowlesi* primarily causes chronic infection in long-tailed and pig-tailed macaques, resulting in several life-threatening complications, including renal failure, liver failure, and several non-malarial symptoms. However, in terms of hematological analysis, the clinical manifestation of knowlesi malaria in humans involves hypoglycemia, anemia, and hyperbilirubinemia [5]. *P. falciparum* causes a diffuse encephalopathy called cerebral malaria (CM), which is the principal cause of malaria-related death. Notably, angiopoietin-1 (ANG1) and angiopoietin-2 (ANG2), which are major regulators of angiogenesis, have been used to identify CM severity [6]. While ANG2 levels were found to be higher in patients with severe malaria, ANG1 levels were lower. Therefore the ratio of ANG2 to ANG1 can be used to assess malaria severity, with a higher ratio indicating more severe malaria [6]. Thus, characterization of these biomarkers in a patient’s serum can predict CM severity and facilitate intervention [7]. *P. vivax*, which displays a unique life cycle, is one of the oldest-known parasites infecting humans [8]. In the primary attack, uninucleate sporozoites (spz) of *P. vivax* are delivered to humans via spz-infected mosquitoes and invade human hepatocytes. Subsequently, the spz either develop into merozoites within infected hepatocytes or remain in a dormant stage as hypnozoites. Activation of dormant hypnozoites following a primary attack can result in an additional blood stage called a relapse [9].

The ability to accurately measure and compare protein expression levels is one of the most important goals in post-genomics malaria research. Earlier studies have utilized two-dimensional gel electrophoresis to analyze malarial proteins [10]. In addition, the combination of two-dimensional gel electrophoresis with mass spectrometry (MS) is a well-established technique for monitoring altered expression of proteins within complex mixtures [11,12,13]. However, this technique has some disadvantages, including difficulties associated with reproducibility, detection of scarce proteins, and analysis of proteins with high molecular weights or isoelectric points [14]. Therefore, in the present study, we have made use of the isobaric tags for relative and absolute quantitation (iTRAQ) technique to conduct a quantitative and comparative proteomic analysis of serum from malaria-infected patients and healthy subjects in order to facilitate the identification of novel malarial biomarkers.

## 2. Results and Discussion

### 2.1. Identification of Candidate Biomarkers by iTRAQ

At the UMMC, serum samples were collected from 25 newly diagnosed malaria patients infected with *P. knowlesi* (*n* = 9), *P. vivax* (*n* = 6) or *P. falciparum* (*n* = 10). In addition, 23 samples were obtained randomly from normal healthy individuals. It is known that the identification of potential serum biomarkers can be complicated by the high abundance of proteins in serum samples [15]. Thus, to reduce the wide range of proteins within our samples and to increase the likelihood of MS-based identification of medium/low abundance proteins, we performed albumin depletion with an albumin segregation column (ASKc). In order to identify and quantify differentially expressed proteins in malaria patients relative to controls, albumin depleted sera were pooled, concentrated, and labeled with isobaric tags using iTRAQ. The serum proteins were considered to be upregulated in malaria-infected patients when the malaria to non-malaria iTRAQ ratio was ≥1.5, whereas an iTRAQ ratio ≤0.67 indicated downregulation of a specific serum protein during malaria infection.

### 2.2. Analysis of ASKc-Depleted Sera by iTRAQ

**Figure 1:**
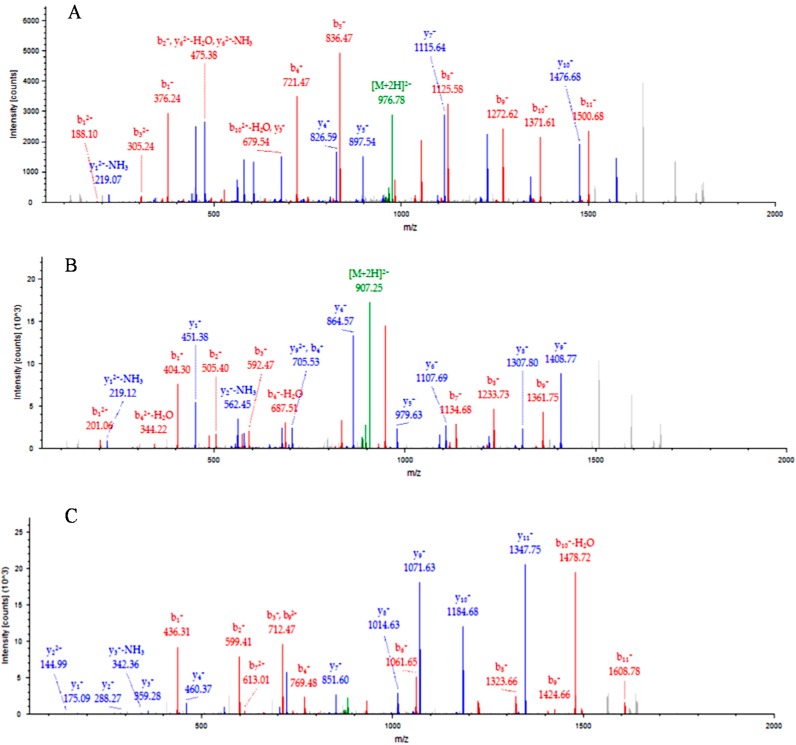
Peptide fragment spectral of the cell adhesion molecule-4 (CADM4) (panel **A**); C-reactive protein isoform 2 (CRP) (panel **B**) and haptoglobin (HAP) (panel **C**).

A total of 152 proteins (≥95% confidence) were detected following albumin depletion (Supplementary Table S1). Two upregulated proteins, namely cell adhesion molecule-4 (CADM4) and C-reactive protein isoform 2 (CRP) were upregulated in the malaria-infected samples compared to the control sera. The iTRAQ ratios for CADM4 and CRP indicated a more than two-fold increase in expression of these serum proteins in malaria-infected samples compared to controls. On the other hand, haptoglobin (HAP) was found to be downregulated. Figure 1 shows the peptide fragment spectral of these proteins. Table 1 demonstrates the iTRAQ ratios for selected serum proteins in malaria-infected patients (*P. knowlesi*, *P.* *falciparum* and *P.* *vivax*) relative to uninfected controls.

**Table 1:** Isobaric tags for relative and absolute quantitation (iTRAQ) ratios for selected serum proteins in malaria-infected patients (*P. knowlesi*, *P.* *falciparum* and *P.* *vivax*) relative to uninfected controls.

Protein	M1/C1	M2/C1	M3/C1
HAP	0.540	0.633	0.435
CRP	3.243	2.154	1.925
CADM4	6.386	3.688	9.760

M1: *P. vivax* malaria serum; M2: *P. falciparum* malaria serum; M3: *P. knowlesi* malaria serum; C1: control serum.

### 2.3. Validation of HAP by Protein Expression

**Figure 2:**
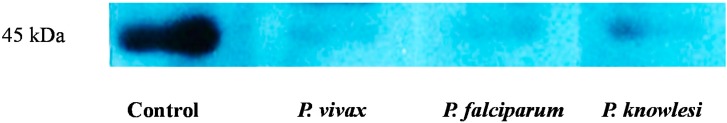
Western blot validation of downregulated HAP in malaria sera.

Downregulated proteins generally serve as effective biomarkers. Therefore, Western Blotting was used to further validate our findings regarding decreased of HAP expression. Upon Western Blotting analysis, HAP was solely present in control sera, but was lacking in three malaria-infected groups (Figure 2). These findings confirmed the present data obtained with iTRAQ.

Unfractionated serum samples from patients infected with *P. vivax*, *P. falciparum*, or *P. knowlesi* were subjected to SDS-PAGE and transferred onto a polyvinylidene difluoride (PVDF) nitrocellulose membrane. Membranes were subsequently immunoblotted with monoclonal antibody against HAP.

In the present study, iTRAQ has been used as a quantitative proteomic technique to analyze sera from patients infected with malaria (*P. knowlesi*, *P.* *falciparum* or *P. vivax*) and non-infected healthy subjects. The present data has validated the expression of CADM4 and HAP in other studies [16,17,18]. The expression levels of CADM4 and CRP were increased two-fold in the sera of malaria patients relative to controls. In contrast, HAP was the only protein that showed lower expression relative to the non-malaria controls. Therefore, the present data proposed that these three serum proteins may represent candidate biomarkers for the development of diagnostic tools for detecting malaria caused by *P. knowlesi*, *P. falciparum* and *P. vivax.*

Although cell adhesion molecules are constitutively expressed in view of its roles as recruiter in immune cells, they may also be upregulated or induced by cytokines and/or foreign antigens [19]. Indeed, previous studies have demonstrated the upregulation of cell adhesion molecules in patients with malaria [20]. In particular, the increased expression of intercellular adhesion molecule-1 was described in patients infected with *P.* *falciparum* [21]. Moreover, others have reported that cell adhesion molecules display significantly increased expression levels during acute malaria infection [22]. If these adhesion molecules were capable of interacting with *Plasmodium* species, they could play an important role in infection or parasite-induced immune regulation. In this regard, a previous study revealed that a primary step during the pathogenesis of *P. falciparum* involves binding to endothelial cells via cell adhesion molecules, as this inducible adhesion molecule is widely distributed on the surface of capillaries [23]. To the best of our knowledge, CADM-4 has never been reported to be altered in the patients infected with malaria diseases.

CRP is a highly conserved molecule, which is a member of the pentraxin family of proteins. It is secreted by the liver in response to a variety of inflammatory cytokines. CRP levels increase rapidly in response to trauma, inflammation, and infection. For this reason, CRP is commonly used to monitor various inflammatory states. While immunoglobulins usually detect specific epitopes on antigens, CRP recognizes altered self or foreign molecules based on pattern recognition [24]. A previous study has indicated that CRP is a sensitive marker for inflammation, and can be used to predict the risk of coronary heart disease [25]. Indeed, inflammatory reactions are often associated with disease states. So far, the detection of malaria is based on the presence of parasitemia and fever. However, some cases of malaria do not involve measurable fever. During afebrile malaria, CRP levels have been highly correlated with parasite density [26]. High levels of CRP have been suggested as an indicative in individuals with high parasite densities [26]. Therefore, our findings indicated that CRP levels were clearly upregulated in malaria-infected individuals, which might be related to the ability of recognition of *Plasmodium* by CRP. In fact, CRP has been suggested to affect hepatic development of *Plasmodium* by preventing sporozoite penetration into hepatocytes, which is mediated by an antibody-like blocking action [27].

HAP is an acute phase protein that is present in most body fluids of humans and other mammals. It binds to free plasma hemoglobin [28], facilitating the degradation of hemoglobin upon intravascular hemolysis [29]. A previous study has demonstrated that hemolysis during malaria infection would lead to a subsequent increase in HAP levels which could reduce disease symptoms due to toxicity to *Plasmodium* parasites [30]. Our results indicated that HAP expression in downregulated in malaria-infected patients relative to healthy control individuals. Indeed, this finding seems contradictory to the role of HAP as a positive acute phase protein. However, low HAP expression in malaria-infected patients may result in higher *Plasmodium* parasitemia, which was in agreement with the findings of Hurt *et al.*, 1994 [26]. Similarly, it has been reported that certain concentrations of HAP can be toxic to *P. falciparum* during an acute phase response [31].

## 3. Experimental Section

### 3.1. Sample Collection

Blood was collected from newly diagnosed malaria patients (*P. knowlesi* (*n* = 9), *P. vivax* (*n* = 6) or *P. falciparum* (*n* = 10)) at the University of Malaya Medical Centre (UMMC, Kuala Lumpur, Malaysia). In order to confirm the *Plasmodium* species infecting each patient, samples were screened using conventional light microscopy, *P. falciparum* histidine-rich protein-2 (PfHRP2)-based lateral flow rapid diagnostic kits, and nested polymerase chain reaction (PCR) [32]. Also, control blood samples (*n* = 23) were obtained randomly from normal healthy individuals. All subjects gave their consent for participation, and the study was approved by the Ethical Committee of UMMC in accordance with the International Conference on Harmonization-Good Clinical Practice (ICH-GCP) guidelines and the declaration of Helsinki.

### 3.2. Albumin Depletion

Albumin was depleted using the Albumin Segregation Kit (ITSI Biosciences, Johnstown, PA, USA) according to the manufacturer’s protocol. Briefly, albumin segregation matrix (ASM), contained within a macrospin column, was washed three times with ASM Buffer 1 (included in the kit). The buffer was removed by centrifugation (2000 rpm, 5 s). Serum sample was diluted with ASM Buffer 1 to a final volume of 300 μL and was mixed by gentle vortexing. The diluted serum was then transferred to the ASM-containing macrospin column. Following incubation (1 min at room temperature), the column was centrifuged (2000 rpm, 5 s) without drying the matrix. The flowthrough was again added to the ASM column and was centrifuged. This process was repeated once more, and the final flowthrough was saved. The ASM column was washed with ASM Buffer 1 (300 μL) and centrifuged (2000 rpm, 5 s). The flowthrough from this washing step was combined with the previously saved flowthrough. This combined sample was finally concentrated with a 5-kDa molecular weight cutoff (MWCO) spin column *via* centrifugation (12,000× *g*, 15 min). After the sample was concentrated to less than 25 μL, 300 μL of ASM Buffer 2 (provided in the kit) was added to the spin column. The sample was again concentrated to a volume less than 25 μL. The ToPA™ assay kit (ITSI Biosciences, Johnstown, PA, USA) was used to determine protein concentrations.

### 3.3. Sample Preparation and Labeling for iTRAQ

Serum proteins from control, *P. vivax*, *P. falciparum* and *P. knowlesi* (27 μg each) samples were used in the iTRAQ analysis. Sample preparation and labeling for iTRAQ were performed based on the manufacture’s protocol (ITSI Bioscience, Johnstown, PA, USA). One microliter of 2% sodium dodecyl sulfate (SDS) was added to denature the proteins (mixed by vortexing). Disulfide groups were reduced by incubation at 60 °C for 1 h with 50 mM Tris-(2-carboxyethyl)-phosphine. The samples were then cooled, and the free cysteine groups were blocked with 200 mM methyl methanethiosulfonate in isopropanol (incubated for 10 min at room temperature). Trypsin was then added to the samples (5%, *w*/*w*), which were incubated overnight at 37 °C. Following trypsin digestion, control, *P. vivax*, *P. falciparum*, and *P. knowlesi* samples were labeled with isobaric tags for 2 h (*m*/*z*: 114, 116, 118 and 119, respectively). After labeling, the digested and labeled samples were combined and vortexed. The final mixed sample was cleaned using an SCX column to remove iTRAQ reagents, reducing agents, alkylating agents, and other impurities. The peptides were eluted from the column using 450 mM ammonium acetate buffer (pH 2.0). The eluted peptides were dried using a speedvac to remove the ammonium acetate. Dried peptides were dissolved in 0.1% formic acid containing 5% acetonitrile, and 15 μL of this solution was injected into the LTQ XL™ mass spectrometer (Thermo Scientific, Waltham, MA, USA).

### 3.4. Liquid Chromatography-Tandem MS (LC-MS/MS) Analysis

All LC-MS/MS analyses were performed using the ThermoElectron ProteomeX Workstation (Thermo Corp., San Jose, CA, USA), which includes a high-performance liquid chromatography HPLC front end and an LTQ XL™ nano-spray-ion-trap mass spectrometer back end. The dried, trypsin-digested peptides were re-suspended in 20 μL of 0.1% formic acid containing 5% acetonitrile. Subsequently, 15 μL of the mixture was loaded onto a Thermo PicoFrit C18 nanospray column and analyzed by HPLC. The peptides were eluted from the column using a linear acetonitrile gradient (2%–45% over 230 min) into the LTQ XL™ mass spectrometer via a nanospray source (spray voltage: 1.8 kV; ion transfer capillary: 180 °C). A data-dependent Top 3 method was used for peptide identification, involving a full MS scan (*m*/*z*: 400–1500) followed by MS/MS scans on the three most abundant ions. Each MS/MS analysis was followed by pulsed Q dissociation (PQD) activation for iTRAQ reporter ion fragmentation, allowing for peptide quantitation.

### 3.5. Data Analysis

Protein identification and the number of missed cleavages were determined using Proteome Discoverer 1.3 software (Thermo Scientific, Waltham, MA, USA). Search parameters were set for trypsin digestion, allowing for up to two missed cleavages per peptide. In addition, static modification C 57.0215, N-term 144, and K 144 were set as fixed modifications. Oxidation of methionine was used as variable modification. Peptides were filtered subject to the default charge *vs.* XCfilter: 1 + 1.50, 2 + 2.00, and 3 + 2.50. Moreover, precursor and fragment ion peaks were searched with a mass tolerance of 5000 ppm and 2 Da, respectively.

### 3.6. Western Blotting

Sera from the control individuals and each of the three malaria patients were separated by SDS-polyacrylamide gel electrophoresis (SDS-PAGE) using a 12.5% gel. Western blotting was performed following electrophoretic transfer of the proteins onto polyvinylidene difluoride (PVDF) membranes as previously described [13,14]. Membranes were probed overnight using a monoclonal antibody against haptoglobin (anti-HAP, Sigma Aldrich, St. Louis, MO, USA), followed by incubation (1 h at room temperature, 1:5000) with monoclonal anti-rat IgG conjugated to horseradish peroxidase (HRP, Invitrogen, Carlsbad, CA, USA). The results were visualized using chemiluminescent reagent (Pierce, Rockford, IL, USA) and X-ray film (18 × 24 cm, Kodak).

## 4. Conclusions

In summary, the data of this study suggest that HAP, CRP, and CADM4 could be used as panel biomarkers of *P. vivax*, *P. falciparum* or *P. knowlesi* infection. However, further validation of these findings will need to be carried out in more extensive clinically representative populations.

## Supplementary Materials

Supplementary table can be found at http://www.mdpi.com/1422-0067/15/11/19952/s1.
